# Supplementary material for: Analyzing the innovation ability of listed companies in the core area of the Huaihai economic zone
Source: PLoS One. 2023 May 2;18(5):e0285181. doi: 10.1371/journal.pone.0285181 (PMC10153750; doi:10.1371/journal.pone.0285181)
Supplement: S1 Data — (DOCX) [file pone.0285181.s001.docx]

Appendix

Log in to CSMAR database, select the data center, and then select the company research series. First, set the time through the financial statement module. Below is the code setting - code screening, which can filter the stock, industry, regional classification, and so on. Make a good choice and then download the data; Then, through the basic information module of the company, choose to download the basic information data of the listed companies in the corresponding cities, and finally integrate the data, and then use the calculation formula set by the indicator system to calculate the original data

Appendix : Original data

| Company abbreviation | particular year | Total assets | R&D investment intensity | R&D investment | Proportion of R&D personnel | Quantity of R&D personnel | Proportion of employees with  Bachelor’s degree or above | Intangible assets | Growth rate of intangible  assets率 | Income growth rate | Net profit growth rate | ROE | Gross operating margin |
| --- | --- | --- | --- | --- | --- | --- | --- | --- | --- | --- | --- | --- | --- |
| XCMG | 2017 | 4977002.54 | 5.607433 | 163350.7271 | 24.49026 | 2162 | 64.81645 | 198310.7 | 8.93021 | 72.4629 | 374.9803 | 4.6198 | 18.8869 |
| XCMG | 2018 | 6124988.23 | 4.537816 | 201524.4449 | 24.82455 | 2370 | 66.67831 | 220225.9 | 11.05095 | 52.44875 | 99.81792 | 7.5581 | 16.6889 |
| XCMG | 2019 | 7729165.71 | 4.150913 | 245634.4359 | 26.41164 | 2596 | 68.02547 | 239698.9 | 8.842321 | 33.24925 | 77.3344 | 11.3942 | 17.4532 |
| XCMG | 2020 | 9179717.67 | 5.072448 | 375199.6088 | 22.85853 | 2818 | 81.15331 | 249351.1 | 4.0268 | 24.99687 | 2.751614 | 11.0465 | 17.0709 |
| XCMG | 2021 | 11002910.7 | 5.279897 | 445240.9166 | 22.97414 | 2923 | 82.174 | 311971.2 | 25.11321 | 14.00526 | 50.76615 | 15.8319 | 16.2422 |
| **Grand** Industrial **Holding** | 2017 | 900497.078 | 52 | 5279314.614 | 32.45638 | 242 | 95.51282 | 1802.01 | -59.9552 | 34.02301 | -167.27 | -12.7081 | 0.6621 |
| **Grand** Industrial **Holding** | 2018 | 695639.142 | 5.7 | 376353.4212 | 33.57834 | 174 | 92.32143 | 1599.064 | -11.2622 | -34.965 | -125.051 | 3.481 | 0.7277 |
| **Grand** Industrial **Holding** | 2019 | 685302.786 | 5.2 | 352321.6759 | 35.03163 | 199 | 88.49145 | 5875.733 | 267.4482 | 2.615982 | 110.772 | 7.0705 | 1.4024 |
| **Grand** Industrial **Holding** | 2020 | 661408.652 | 5.3 | 312919.7134 | 34.86101 | 193 | 88.92456 | 5563.992 | -5.30557 | -12.8593 | -72.3913 | 1.9101 | 1.3468 |
| **Grand** Industrial **Holding** | 2021 | 762888.031 | 5.9 | 481215.2594 | 40.74716 | 251 | 76.07891 | 9088.149 | 63.33864 | 38.14346 | 423.152 | 9.5932 | 1.2063 |
| **Shantui** Construction Machinery | 2017 | 956102.984 | 3.40868 | 21,647.84 | 34.15771 | 953 | 50.99616 | 58049 | -12.8666 | 44.19017 | 251.9547 | 1.7429 | 16.6218 |
| **Shantui** Construction Machinery | 2018 | 937185.202 | 2.857388 | 22864.03595 | 33.00071 | 926 | 52.05937 | 61143.64 | 5.331079 | 25.99557 | 36.90105 | 2.3335 | 15.9613 |
| **Shantui** Construction Machinery | 2019 | 893079.819 | 3.593758 | 23011.24475 | 36.00982 | 1027 | 53.02101 | 62800.78 | 2.710237 | -19.9783 | -27.1852 | 1.6762 | 15.6634 |
| **Shantui** Construction Machinery | 2020 | 970268.656 | 3.544185 | 25157.19595 | 38.05455 | 1158 | 55.51907 | 63098.87 | 0.474655 | 10.85483 | 69.96697 | 2.7923 | 13.7339 |
| **Shantui** Construction Machinery | 2021 | 1074633.25 | 3.710531 | 33988.25622 | 37.75273 | 1139 | 58.87978 | 60751.34 | -3.72039 | 29.04669 | 100.4397 | 4.9505 | 14.3524 |
| Henan **Shenhuo** Coal﹠Power | 2017 | 5393221.98 | 0.432927 | 8181.961023 | 7.179403 | 435 | 22.9412 | 528408.6 | 5.521407 | 11.814 | -129.912 | 3.9577 | 24.0126 |
| Henan **Shenhuo** Coal﹠Power | 2018 | 5346854.32 | 0.328251 | 6182.529146 | 6.95553 | 427 | 23.11893 | 566343.3 | 7.179042 | -0.34063 | -120.863 | -0.8257 | 12.6255 |
| Henan **Shenhuo** Coal﹠Power | 2019 | 4939358.41 | 0.302517 | 5329.693014 | 6.139453 | 420 | 25.98868 | 488464.8 | -13.7511 | -6.46114 | -990.3 | 6.9052 | 14.8018 |
| Henan **Shenhuo** Coal﹠Power | 2020 | 6063729.05 | 0.718204 | 13508.86544 | 5.998087 | 439 | 27.84372 | 463245.8 | -5.16291 | 6.762451 | -55.7588 | 2.3691 | 21.4983 |
| Henan **Shenhuo** Coal﹠Power | 2021 | 5352794.75 | 0.462259 | 15925.56111 | 20.239 | 1338 | 29.12207 | 480946 | 3.820921 | 83.16305 | 1110.267 | 22.7463 | 35.6851 |
| **Huafu** Fashion | 2017 | 1603115.16 | 1.631145 | 20546.77662 | 31.17871 | 984 | 12.76389 | 59972.7 | 1.334003 | 42.54465 | 37.0868 | 12.2461 | 11.1897 |
| **Huafu** Fashion | 2018 | 1846232.28 | 1.345611 | 19252.23662 | 30.81336 | 913 | 12.97911 | 63057.53 | 5.143729 | 13.58226 | 7.240451 | 10.369 | 10.5187 |
| **Huafu** Fashion | 2019 | 1833587.55 | 1.023159 | 16254.79884 | 33.93006 | 815 | 12.58316 | 79904.44 | 26.71673 | 11.03928 | -45.4942 | 5.8783 | 7.8599 |
| **Huafu** Fashion | 2020 | 1761007.56 | 1.07592 | 15312.19884 | 37.44292 | 738 | 12.39155 | 88634.23 | 10.92528 | -10.4183 | -214.455 | -7.4204 | 1.5146 |
| **Huafu** Fashion | 2021 | 2013818.59 | 0.919064 | 15356.1242 | 39.64592 | 739 | 11.25196 | 93900.74 | 5.941849 | 17.40273 | -230.508 | 9.0762 | 9.6383 |
| Shandong **Sun paper** | 2017 | 2605627.84 | 2.516312 | 47543.92066 | 32.37166 | 763 | 31.20201 | 66363.68 | 19.24766 | 30.70665 | 94.27743 | 23.4828 | 26.0321 |
| Shandong **Sun paper** | 2018 | 2952313.87 | 2.280533 | 49643.55169 | 32.27612 | 865 | 32.82303 | 85993.82 | 29.57964 | 15.21153 | -0.35259 | 19.1212 | 23.4518 |
| Shandong **Sun paper** | 2019 | 3229517.24 | 2.322165 | 52858.74719 | 23.98783 | 1025 | 2946.897 | 86150.4 | 0.182092 | 4.567658 | -1.99066 | 16.0827 | 22.5429 |
| Shandong **Sun paper** | 2020 | 3586634.01 | 2.46091 | 53127.71782 | 27.81699 | 1143 | 36.68095 | 102322.2 | 18.77162 | -5.15781 | -10.4527 | 12.7211 | 19.4377 |
| Shandong **Sun paper** | 2021 | 4273747.23 | 1.681988 | 53817.97459 | 28.76942 | 1370 | 35.80451 | 127406.4 | 24.51489 | 48.2105 | 50.75687 | 16.927 | 17.3723 |
| Shandong **Ruyi** Woolen Garment | 2017 | 458487.188 | 3.271292 | 3903.391134 | 32.40034 | 382 | 28.34135 | 18942.93 | 12.05553 | 31.68478 | 154.4151 | 3.252 | 21.1477 |
| Shandong **Ruyi** Woolen Garment | 2018 | 479617.052 | 3.375672 | 4482.387465 | 32.48869 | 359 | 27.51494 | 17931.38 | -5.33998 | 11.28238 | 34.02525 | 4.1606 | 24.7022 |
| Shandong **Ruyi** Woolen Garment | 2019 | 488876.722 | 3.877087 | 4459.014637 | 15.2696 | 337 | 72.88639 | 17275.35 | -3.65857 | -13.3868 | -59.1577 | 1.6585 | 26.2206 |
| Shandong **Ruyi** Woolen Garment | 2020 | 491187.162 | 3.400216 | 2673.402202 | 13.67781 | 270 | 79.62888 | 16490.59 | -4.54267 | -31.6365 | -100.72 | -0.0118 | 26.8861 |
| Shandong **Ruyi** Woolen Garment | 2021 | 480734.324 | 4.779697 | 3296.779002 | 23.17597 | 270 | 47.60932 | 15712.08 | -4.72094 | -12.2733 | 1495.545 | -0.1874 | 28.454 |
| Jiangsu **Nhwa** Pharmaceutical | 2017 | 330156.948 | 3.422639 | 11615.33124 | 8.62069 | 270 | 82.57316 | 6672.191 | -14.5221 | 12.45586 | 24.38502 | 16.3678 | 47.9671 |
| Jiangsu **Nhwa** Pharmaceutical | 2018 | 403016.532 | 4.458724 | 17202.50756 | 8.476027 | 297 | 84.0691 | 11096.21 | 66.30536 | 13.68697 | 38.35011 | 19.1685 | 56.1451 |
| Jiangsu **Nhwa** Pharmaceutical | 2019 | 420150.193 | 4.554347 | 18897.40776 | 9.710799 | 366 | 85.3874 | 10619.06 | -4.30013 | 7.546167 | 26.56452 | 20.0318 | 62.615 |
| Jiangsu **Nhwa** Pharmaceutical | 2020 | 474844.026 | 7.934066 | 26668.80464 | 15.25553 | 600 | 88.36217 | 10103.84 | -4.85183 | -18.9913 | 10.65785 | 18.8605 | 75.5421 |
| Jiangsu **Nhwa** Pharmaceutical | 2021 | 569718.671 | 10.22487 | 40241.63927 | 18.02298 | 753 | 88.29248 | 9598.22 | -5.00426 | 17.08744 | 9.753369 | 17.7156 | 77.2 |
| Jiangsu **Yanghe** Brewery Joint-Stock | 2017 | 4325814.07 | 0.189817 | 3780.76348 | 4.517134 | 348 | 51.27113 | 165354.6 | 1.157549 | 15.91582 | 14.0201 | 23.8378 | 66.4566 |
| Jiangsu **Yanghe** Brewery Joint-Stock | 2018 | 4956376.78 | 0.136094 | 3288.011063 | 4.94713 | 393 | 51.95553 | 178196.2 | 7.766051 | 21.29668 | 22.61055 | 25.7142 | 73.7033 |
| Jiangsu **Yanghe** Brewery Joint-Stock | 2019 | 5345503.78 | 0.721757 | 16691.70253 | 4.700297 | 396 | 53.32616 | 174713.4 | -1.95445 | -4.27704 | -8.98594 | 21.0682 | 71.3473 |
| Jiangsu **Yanghe** Brewery Joint-Stock | 2020 | 5386625.93 | 1.276525 | 26936.01456 | 6.453509 | 549 | 53.73634 | 171352.7 | -1.92358 | -8.75804 | 1.333796 | 19.9693 | 72.2672 |
| Jiangsu **Yanghe** Brewery Joint-Stock | 2021 | 6779870.42 | 1.067933 | 27072.30017 | 6.33539 | 575 | 50.54578 | 167959.8 | -1.98005 | 20.13704 | 0.376921 | 18.5614 | 75.324 |
| Shandong **Yabo** Technology | 2017 | 265639.016 | 2.690204 | 3705.100548 | 63.51351 | 141 | 40 | 5972.784 | -1.69814 | 7.155763 | 10.64325 | 35.7064 | 38.1008 |
| Shandong **Yabo** Technology | 2018 | 164432.578 | 10.82183 | 2482.057554 | 56.77419 | 88 | 41.00529 | 5869.606 | -1.72747 | -83.3469 | -274.026 | -75.0527 | -34.1487 |
| Shandong **Yabo** Technology | 2019 | 153432.001 | 5.074701 | 531.107936 | 83.33333 | 30 | 41.37931 | 4902.456 | -16.4772 | -54.3689 | -30.8431 | -142.06 | -20.9073 |
| Shandong **Yabo** Technology | 2020 | 140944.349 | 2.445506 | 306.604315 | 63.63636 | 28 | 38.59649 | 3307.046 | -32.5431 | 19.79456 | -89.3018 | -51.0483 | 3.9059 |
| Shandong **Yabo** Technology | 2021 | 99876.1427 | 6.047074 | 757.233189 | 60 | 57 | 51.07527 | 445.0608 | -86.542 | -0.12084 | 2793.791 | -284.418 | 34.7685 |
| LFBC | 2017 | 403240.353 | 3.115518 | 5765.705321 | 12.07835 | 111 | 45.36032 | 10533.77 | -3.98259 | 27.04436 | -66.928 | 1.4055 | 30.4457 |
| LFBC | 2018 | 352686.999 | 3.055508 | 4526.405939 | 15.59278 | 121 | 38.33992 | 8731.164 | -17.1126 | -19.9525 | -2568.35 | -41.7609 | 24.336 |
| LFBC | 2019 | 270268.808 | 2.315619 | 3483.697264 | 43.04813 | 322 | 40.67428 | 8044.287 | -7.86696 | 1.555465 | -40.9619 | -36.9964 | 22.2122 |
| LFBC | 2020 | 231104.922 | 1.183996 | 1588.88864 | 17.24138 | 95 | 36.32169 | 4945.654 | -38.5197 | -10.799 | -102.87 | 1.2917 | 13.7245 |
| LFBC | 2021 | 172794.996 | 0.89454 | 1291.655127 | 17.28625 | 93 | 38.31909 | 4685.515 | -5.25995 | 7.597874 | -3413.59 | -53.7837 | -0.3275 |
| Shandong Sacred Sun Power Sources | 2017 | 203188.975 | 3.263563 | 5569.166656 | 33.64706 | 286 | 42.26753 | 6288.202 | 0.555489 | 9.977327 | -41.2415 | 2.6569 | 18.241 |
| Shandong Sacred Sun Power Sources | 2018 | 191264.153 | 3.17624 | 5830.177039 | 34.57249 | 279 | 45.54176 | 6003.598 | -4.526 | 7.564824 | -44.404 | 1.4712 | 16.0597 |
| Shandong Sacred Sun Power Sources | 2019 | 191148.552 | 3.298147 | 6123.160382 | 35.03106 | 282 | 46.15826 | 5874.539 | -2.14971 | 1.143289 | 37.7421 | 1.995 | 18.9914 |
| Shandong Sacred Sun Power Sources | 2020 | 241327.898 | 3.832164 | 6749.546715 | 52.38938 | 296 | 29.38118 | 5809.427 | -1.10836 | -5.13085 | 19.62704 | 1.9623 | 16.8248 |
| Shandong Sacred Sun Power Sources | 2021 | 253187.623 | 3.203789 | 6697.599936 | 14.39842 | 292 | 100 | 5743.584 | -1.1334 | 18.6929 | 18.01019 | 1.9659 | 14.3209 |
| Jiangsu **Shuangxing** Color Plastic New Materials | 2017 | 905024.008 | 3.195685 | 9660.413955 | 27.82609 | 128 | 37.76683 | 9541.003 | -2.34152 | 18.42002 | -43.8809 | 1.1721 | 12.0026 |
| Jiangsu **Shuangxing** Color Plastic New Materials | 2018 | 930266.761 | 3.149869 | 12150.82671 | 30.59361 | 134 | 32.80899 | 12743.08 | 33.56119 | 27.60908 | 330.4668 | 4.2583 | 17.2779 |
| Jiangsu **Shuangxing** Color Plastic New Materials | 2019 | 899541.809 | 3.246732 | 14520.37958 | 29.37365 | 136 | 32.30984 | 15201.79 | 19.29454 | 15.93599 | -46.013 | 2.2612 | 12.9397 |
| Jiangsu **Shuangxing** Color Plastic New Materials | 2020 | 1021779.8 | 3.10725 | 15726.76851 | 29.66418 | 159 | 33.6895 | 33433.14 | 119.9289 | 13.1701 | 315.3303 | 9.0328 | 22.5474 |
| Jiangsu **Shuangxing** Color Plastic New Materials | 2021 | 1212530.55 | 4.499754 | 26688.96609 | 61.3913 | 353 | 27.76437 | 34693.19 | 3.768865 | 17.18707 | 92.27637 | 15.6464 | 32.8592 |
| **Limin** Group | 2017 | 238588.093 | 4.10929 | 5849.248334 | 60.34483 | 280 | 24.90607 | 16002.12 | 198.0219 | 22.66376 | 25.21403 | 8.5396 | 28.0162 |
| **Limin** Group | 2018 | 273531.448 | 3.761608 | 5714.567991 | 64.84211 | 308 | 24.52246 | 15662.41 | -2.12289 | 6.727564 | 47.53374 | 11.5979 | 27.165 |
| **Limin** Group | 2019 | 476728.626 | 4.786079 | 13557.40481 | 31.60055 | 462 | 38.19227 | 35660.13 | 127.6797 | 86.46051 | 65.46654 | 16.5894 | 26.6998 |
| **Limin** Group | 2020 | 518971.709 | 4.228929 | 18556.74357 | 45.03393 | 730 | 38.1861 | 38327.55 | 7.480117 | 54.9083 | 24.19748 | 17.8663 | 24.7401 |
| **Limin** Group | 2021 | 688329.256 | 4.003599 | 18967.31559 | 37.84057 | 750 | 43.28456 | 48546.61 | 26.66243 | 7.96523 | -22.0613 | 12.4184 | 22.6044 |
| Shandong **Fengyuan** Chemical | 2017 | 75145.7604 | 3.615732 | 1160.32145 | 48.42105 | 46 | 22.89157 | 3363.996 | 13.46637 | 25.14685 | 67.05046 | 6.8383 | 23.3927 |
| Shandong **Fengyuan** Chemical | 2018 | 78261.489 | 5.144189 | 1362.237026 | 41.73913 | 48 | 24.52026 | 3274.235 | -2.66828 | -17.4811 | -56.9706 | 2.834 | 21.0219 |
| Shandong **Fengyuan** Chemical | 2019 | 92780.5232 | 2.270369 | 1039.75155 | 30.12821 | 47 | 34.43709 | 3179.483 | -2.89388 | 72.94078 | -30.5425 | 1.944 | 14.4179 |
| Shandong **Fengyuan** Chemical | 2020 | 110698.705 | 4.979871 | 1779.128168 | 42.99065 | 46 | 25.84541 | 3085.152 | -2.96686 | -21.989 | -357.801 | -5.159 | 8.8038 |
| Shandong **Fengyuan** Chemical | 2021 | 181682.516 | 3.55 | 2850.819374 | 37.58865 | 53 | 30.45356 | 2990.439 | -3.06996 | 124.7772 | -281.66 | 6.4083 | 15.2641 |
| Shandong **Liancheng** Precision Manufacturing | 2017 | 114994.112 | 1.148676 | 700.335185 | 12.14575 | 30 | 13.21562 | 3887.031 | 29.98194 | -24.0781 | -14.9342 | 8.4013 | 25.986 |
| Shandong **Liancheng** Precision Manufacturing | 2018 | 122421.067 | 2.568458 | 1709.012089 | 31.83673 | 78 | 13.61868 | 7271.064 | 87.05961 | 9.13505 | -1.22795 | 6.7193 | 24.0575 |
| Shandong **Liancheng** Precision Manufacturing | 2019 | 125804.773 | 2.691262 | 2012.632333 | 55.55556 | 125 | 13.24308 | 6953.144 | -4.3724 | 12.39212 | 2.483942 | 6.6414 | 25.8438 |
| Shandong **Liancheng** Precision Manufacturing | 2020 | 151482.761 | 2.704158 | 2467.59232 | 56.52174 | 156 | 15.79851 | 6789.379 | -2.35527 | 22.02051 | 56.88562 | 9.2187 | 26.9869 |
| Shandong **Liancheng** Precision Manufacturing | 2021 | 183516.482 | 2.295325 | 2778.663887 | 45.45455 | 160 | 15.26453 | 6887.391 | 1.443597 | 32.66322 | -0.39577 | 8.0111 | 21.5492 |
| Jiangsu **Xiuqiang** Glasswork | 2017 | 216491.263 | 3.283504 | 4523.975506 | 11.54625 | 171 | 43.26614 | 3362.509 | -5.69217 | 20.09397 | -2.2644 | 9.1331 | 31.6729 |
| Jiangsu **Xiuqiang** Glasswork | 2018 | 168865.347 | 3.136663 | 4393.493073 | 13.64353 | 173 | 42.67923 | 3658.321 | 8.797369 | 1.662174 | -296.738 | -20.7809 | 29.1899 |
| Jiangsu **Xiuqiang** Glasswork | 2019 | 171064.982 | 3.259369 | 4136.313236 | 56.8323 | 183 | 18.71005 | 3181.424 | -13.036 | -9.39798 | -152.281 | 12.7663 | 33.2269 |
| Jiangsu **Xiuqiang** Glasswork | 2020 | 174133.707 | 3.498398 | 4500.121264 | 61.2426 | 207 | 16.95085 | 3092.42 | -2.7976 | 1.361959 | 1.516632 | 12.1343 | 26.4691 |
| Jiangsu **Xiuqiang** Glasswork | 2021 | 189515.562 | 4.114567 | 5998.21592 | 97.47191 | 347 | 13.54642 | 5282.843 | 70.832 | 13.32952 | 12.52539 | 12.52 | 21.8819 |
| Jiangsu **Yunyi** Electric | 2017 | 207216.493 | 7.366875 | 4726.739558 | 228.3465 | 290 | 18.89881 | 3644.134 | -3.00142 | 19.78875 | 28.18437 | 8.0688 | 35.9818 |
| Jiangsu **Yunyi** Electric | 2018 | 222287.419 | 8.685652 | 5721.781811 | 218.9781 | 300 | 17.72316 | 3487.153 | -4.30776 | 2.671642 | -6.07565 | 7.0306 | 35.7525 |
| Jiangsu **Yunyi** Electric | 2019 | 266448.111 | 11.08141 | 7913.934352 | 188.7701 | 353 | 19.72574 | 3422.835 | -1.84442 | 8.409789 | 1.257619 | 6.6609 | 33.0491 |
| Jiangsu **Yunyi** Electric | 2020 | 289944.421 | 9.849253 | 8269.050016 | 182.2581 | 339 | 19.45607 | 2676.622 | -21.801 | 17.55876 | 53.30201 | 9.5133 | 33.6781 |
| Jiangsu **Yunyi** Electric | 2021 | 312209.93 | 9.599925 | 10553.59903 | 156.8627 | 400 | 20.58111 | 2826.753 | 5.60899 | 30.94244 | 20.55627 | 10.4587 | 32.7789 |
| Jiangsu **Wuyang** Parking Industry Group | 2017 | 198636.249 | 4.898003 | 3695.899743 | 24.94457 | 225 | 65.03244 | 11574.26 | 43.37533 | 24.38818 | -1.24688 | 5.3778 | 35.2114 |
| Jiangsu **Wuyang** Parking Industry Group | 2018 | 220263.18 | 5.911172 | 5923.445597 | 25.6785 | 246 | 64.2953 | 11095.99 | -4.13225 | 32.80051 | 84.53244 | 8.497 | 34.9988 |
| Jiangsu **Wuyang** Parking Industry Group | 2019 | 280180.82 | 5.656456 | 7304.622198 | 26.75097 | 275 | 63.1062 | 10469.08 | -5.64982 | 28.8702 | 18.27578 | 9.0531 | 34.2936 |
| Jiangsu **Wuyang** Parking Industry Group | 2020 | 394835.141 | 6.274464 | 10237.18814 | 28.15622 | 310 | 65.1094 | 19387.14 | 85.18469 | 26.34286 | -1.31048 | 6.6826 | 31.7619 |
| Jiangsu **Wuyang** Parking Industry Group | 2021 | 394469.207 | 8.78248 | 13665.1466 | 27.63485 | 333 | 65.31165 | 17616.6 | -9.13258 | -4.63411 | -218.795 | -6.9145 | 16.7149 |
| **Saimo** Technology | 2017 | 178934.884 | 7.796246 | 3561.114141 | 42.2619 | 284 | 63.81766 | 11534.38 | -0.31832 | 37.82571 | -55.0776 | 2.0144 | 43.1342 |
| **Saimo** Technology | 2018 | 162102.551 | 11.64502 | 5005.409109 | 41.53627 | 292 | 68.38521 | 10673.99 | -7.45928 | -5.89798 | -949.342 | -16.6858 | 42.8458 |
| **Saimo** Technology | 2019 | 126634.496 | 10.62369 | 5316.828803 | 40.60742 | 361 | 74.89469 | 9748.157 | -8.67377 | 16.43355 | -2.63576 | -20.6352 | 38.9843 |
| **Saimo** Technology | 2020 | 119712.748 | 11.0051 | 5998.342428 | 34.27835 | 266 | 74.18738 | 8413.122 | -13.6953 | 8.90804 | -104.965 | 1.231 | 38.2382 |
| **Saimo** Technology | 2021 | 134166.219 | 8.516364 | 5002.65956 | 34.75 | 278 | 75.0469 | 7572.421 | -9.99274 | 7.772852 | -267.779 | -2.1036 | 35.3201 |
| Jiangsu **Huaxin** New Material | 2017 | 56340.4389 | 3.128932 | 888.112309 | 28.97727 | 51 | 48.61878 | 2711.056 | -64.1983 | -51.6802 | -346.655 | 11.2184 | 29.5811 |
| Jiangsu **Huaxin** New Material | 2018 | 59725.1379 | 3.153431 | 966.835135 | 27.27273 | 54 | 49.87406 | 2648.489 | -2.30783 | 8.018296 | 8.343154 | 8.9333 | 26.9938 |
| Jiangsu **Huaxin** New Material | 2019 | 65675.551 | 3.291084 | 1038.952227 | 26.57005 | 55 | 51.75 | 2585.767 | -2.36823 | 2.964524 | 10.75425 | 9.1951 | 29.2216 |
| Jiangsu **Huaxin** New Material | 2020 | 66697.1952 | 3.108221 | 818.758141 | 25.88832 | 51 | 52.1164 | 2575.135 | -0.41117 | -16.5576 | -29.8507 | 6.1176 | 26.4607 |
| Jiangsu **Huaxin** New Material | 2021 | 69010.6247 | 3.937769 | 1274.156057 | 24.87805 | 51 | 53.94737 | 2507.16 | -2.63967 | 22.83687 | -42.1965 | 3.4502 | 19.5064 |
| Shandong **Taihe** Technologies | 2019 | 194453.489 | 3.745912 | 4664.487742 | 100 | 111 | 19.71581 | 6984.28 | 178.5733 | 284.8345 | 718.5605 | 13.9975 | 29.9781 |
| Shandong **Taihe** Technologies | 2020 | 212422.147 | 4.279199 | 6542.422969 | 34.68013 | 103 | 49.0099 | 6745.404 | -3.42019 | 22.78059 | 13.73874 | 10.8368 | 22.8078 |
| Shandong **Taihe** Technologies | 2021 | 255620.525 | 3.60969 | 7974.211708 | 33.43849 | 106 | 48.54518 | 6463.026 | -4.18623 | 44.49132 | 42.62896 | 13.9743 | 22.2287 |
| Jiangsu **Sidike** New Materials Science and Technology | 2019 | 208990.128 | 5.779784 | 8280.672266 | 84.55882 | 115 | 13.57285 | 5975.03 | -7.55059 | -35.1461 | -60.0976 | 12.587 | 25.9213 |
| Jiangsu **Sidike** New Materials Science and Technology | 2020 | 320297.043 | 6.157844 | 9479.749206 | 70.5 | 141 | 18.11594 | 17196.54 | 187.8067 | 7.451921 | 62.64104 | 15.4409 | 25.1313 |
| Jiangsu **Sidike** New Materials Science and Technology | 2021 | 459063.395 | 5.493601 | 10900.17294 | 27.62186 | 187 | 48.60014 | 18659.57 | 8.507697 | 28.88669 | 15.34437 | 15.1357 | 26.9596 |
| **Yankuang** Energy Group Company | 2017 | 19488729.1 | 0.015719 | 2377.2 | 11.93969 | 2922 | 35.97384 | 4747692 | 81.96712 | 48.28838 | 242.9151 | 12.2523 | 16.6238 |
| **Yankuang** Energy Group Company | 2018 | 20367990 | 0.096927 | 15800 | 11.55507 | 2944 | 39.51732 | 4517772 | -4.84278 | 7.790035 | 35.53958 | 13.1364 | 19.4895 |
| **Yankuang** Energy Group Company | 2019 | 20782136.3 | 0.132073 | 26500 | 10.49733 | 2592 | 40.31808 | 4931388 | 9.155321 | 23.09004 | 4.257946 | 13.1883 | 13.9106 |
| **Yankuang** Energy Group Company | 2020 | 25891004.1 | 0.237218 | 51000 | 9.66421 | 2596 | 42.05796 | 5885504 | 19.3478 | 7.149181 | -38.4528 | 8.374 | 13.2195 |
| **Yankuang** Energy Group Company | 2021 | 28869554.2 | 0.750045 | 114000 | 12.2329 | 3349 | 44.25352 | 6216678 | 5.626956 | -29.3039 | 171.5361 | 21.0664 | 29.38 |
| Jiangsu **Hengrui** Pharmaceuticals | 2017 | 1803938.48 | 12.7145 | 175913.1108 | 25.12755 | 2167 | 58.01938 | 27925.55 | -1.91778 | 24.71582 | 25.00796 | 22.8538 | 86.6296 |
| Jiangsu **Hengrui** Pharmaceuticals | 2018 | 2236122.96 | 15.33182 | 267048.0551 | 26.57116 | 3116 | 55.80034 | 27268.24 | -2.35378 | 25.89164 | 23.32954 | 22.7257 | 86.5967 |
| Jiangsu **Hengrui** Pharmaceuticals | 2019 | 2755647.55 | 16.73067 | 389633.5999 | 24.07666 | 3442 | 58.51582 | 34976.15 | 28.26698 | 33.70484 | 31.15514 | 23.8132 | 87.492 |
| Jiangsu **Hengrui** Pharmaceuticals | 2020 | 3472958.99 | 17.98821 | 498895.8232 | 26.86813 | 4721 | 60.793 | 34125.29 | -2.4327 | 19.091 | 18.44457 | 22.6431 | 87.9259 |
| Jiangsu **Hengrui** Pharmaceuticals | 2021 | 3926622.17 | 23.94581 | 620328.8327 | 36.18708 | 5478 | 61.81046 | 44245.42 | 29.65582 | -6.59491 | -28.9253 | 13.5145 | 85.556 |
| VVFB | 2017 | 823624.713 | 0.1514 | 703.48685 | 55.20362 | 122 | 10.02722 | 84621.33 | -0.46793 | 4.098698 | -315.642 | 1.6494 | 23.4385 |
| VVFB | 2018 | 835317.817 | 0.108931 | 548.238388 | 77.29469 | 160 | 10.19704 | 68051.44 | -19.5812 | 8.31529 | -35.5035 | 1.123 | 23.0552 |
| VVFB | 2019 | 865311.27 | 0.036162 | 182.227872 | 71.42857 | 150 | 10.76923 | 61249.21 | -9.99572 | 0.124114 | 36.84663 | 1.6667 | 23.5361 |
| VVFB | 2020 | 671913.405 | 0.073082 | 350.707511 | 40.08621 | 93 | 7.222914 | 28348.7 | -53.7158 | -4.7696 | 883.4765 | 15.5661 | 21.19 |
| VVFB | 2021 | 515884.855 | 0.073503 | 335.77336 | 12.29236 | 37 | 9.284392 | 80760.4 | 184.8822 | -4.80623 | -49.1135 | 7.3179 | 19.8316 |
| Jiangsu **Kanion** Pharmaceutical | 2017 | 539370.995 | 7.878662 | 25800.23981 | 7.844184 | 294 | 64.38756 | 33356.9 | 6.111581 | 9.146498 | 0.541155 | 11.1241 | 75.7627 |
| Jiangsu **Kanion** Pharmaceutical | 2018 | 560294.996 | 9.190145 | 35141.32145 | 7.723971 | 319 | 65.56596 | 32408.22 | -2.84403 | 16.76816 | 15.7122 | 11.6916 | 76.334 |
| Jiangsu **Kanion** Pharmaceutical | 2019 | 611082.161 | 10.17862 | 46473.50794 | 6.900329 | 378 | 73.05948 | 31754.36 | -2.01756 | 19.40458 | 18.03255 | 12.9457 | 78.1218 |
| Jiangsu **Kanion** Pharmaceutical | 2020 | 611850.838 | 13.04353 | 39547.01091 | 8.352619 | 397 | 73.66708 | 34079.09 | 7.320983 | -33.5948 | -46.9469 | 6.5135 | 72.8988 |
| Jiangsu **Kanion** Pharmaceutical | 2021 | 601801.924 | 13.97165 | 50976.5548 | 11.27022 | 425 | 74.74727 | 28273.21 | -17.0365 | 20.33835 | 18.36488 | 7.3721 | 71.906 |
| Shandong **Lukang** Pharmaceutical | 2017 | 520191.625 | 4.26599 | 11088.54111 | 13.83266 | 410 | 49.63993 | 21908.72 | 12.67936 | 3.739515 | 336.9455 | 6.3188 | 27.291 |
| Shandong **Lukang** Pharmaceutical | 2018 | 656744.85 | 5.069428 | 16879.19057 | 16.71914 | 531 | 50.96277 | 21850.15 | -0.26733 | 28.09676 | 34.51868 | 6.7086 | 31.1481 |
| Shandong **Lukang** Pharmaceutical | 2019 | 708926.824 | 6.183747 | 23082.19008 | 15.93861 | 540 | 55.90759 | 39276.56 | 79.75419 | 12.10697 | -25.7417 | 4.1257 | 28.2819 |
| Shandong **Lukang** Pharmaceutical | 2020 | 727282.484 | 5.747374 | 24168.33643 | 16.59619 | 628 | 61.32901 | 39508.06 | 0.589399 | 12.65539 | 81.29449 | 7.1502 | 23.3081 |
| Shandong **Lukang** Pharmaceutical | 2021 | 778956.299 | 6.363013 | 31119.41468 | 19.19241 | 789 | 66.09325 | 50555.28 | 27.96195 | 16.30311 | -64.2468 | 2.4703 | 23.4424 |
| Anhui **Hengyuan** Coal Industry and Electricity Power | 2017 | 1424571.57 | 1.981009 | 12948.23076 | 33.56665 | 1103 | 23.48317 | 212164.3 | -4.56352 | 41.30408 | 2666.009 | 17.3025 | 40.8733 |
| Anhui **Hengyuan** Coal Industry and Electricity Power | 2018 | 1445102.64 | 6.310132 | 37231.8783 | 79.90954 | 2120 | 19.69562 | 202368.2 | -4.61721 | -9.7281 | 10.16946 | 16.3157 | 45.8214 |
| Anhui **Hengyuan** Coal Industry and Electricity Power | 2019 | 1542276.03 | 4.293128 | 25767.24015 | 61.52783 | 1780 | 20.93494 | 252926.6 | 24.98334 | 1.722622 | -7.92956 | 13.4291 | 44.5555 |
| Anhui **Hengyuan** Coal Industry and Electricity Power | 2020 | 1568488.46 | 3.470784 | 18082.0669 | 61.06376 | 1791 | 20.48327 | 244729.3 | -3.24096 | -13.1987 | -31.5454 | 8.4963 | 37.6645 |
| Anhui **Hengyuan** Coal Industry and Electricity Power | 2021 | 1776681.48 | 4.190107 | 28280.60559 | 56.72572 | 1729 | 20.27405 | 236984.5 | -3.16466 | 29.5517 | 79.37473 | 14.2664 | 42.1315 |
| **Huaibei Mining** Holdings | 2017 | 241889.117 | 1.005632 | 961.888076 | 10.43219 | 70 | 32.60447 | 55653.19 | -4.28095 | 14.43399 | 31.62376 | 7.7218 | 40.9321 |
| **Huaibei Mining** Holdings | 2018 | 5895664.93 | 2.422315 | 132469.6737 | 45.62983 | 5727 | 21.18348 | 926588.1 | 1564.933 | 5617.424 | 3108.418 | 35.6642 | 20.3464 |
| **Huaibei Mining** Holdings | 2019 | 6228089.27 | 2.070452 | 124405.5229 | 54.38395 | 6643 | 21.61411 | 1019642 | 10.04269 | 9.87238 | -8.46243 | 17.0281 | 16.5316 |
| **Huaibei Mining** Holdings | 2020 | 6701056.46 | 2.626317 | 137292.7431 | 58.23219 | 6621 | 21.63077 | 991456 | -2.76434 | -12.9986 | 1.575628 | 15.4182 | 18.1718 |
| **Huaibei Mining** Holdings | 2021 | 7346396.53 | 2.554945 | 165971.5026 | 62.961 | 7136 | 23.5277 | 1292545 | 30.36837 | 24.26576 | 41.65389 | 18.1598 | 19.7668 |
| Jiangsu **Lianyungang** Port | 2017 | 738297.757 | 52 | 67,999.64 | 68 | 786.76 | 29.10692 | 2508.636 | 9.179886 | 12.04278 | 280.9753 | 0.4426 | 26.6299 |
| Jiangsu **Lianyungang** Port | 2018 | 907429.749 | 5.7 | 7,507.17 | 71 | 874.01 | 34.19444 | 126.8395 | -94.9439 | 0.715954 | 27.83032 | 0.5273 | 26.5823 |
| Jiangsu **Lianyungang** Port | 2019 | 946156.147 | 5.2 | 7,408.09 | 84 | 876.96 | 29.60862 | 111.8187 | -11.8423 | 8.168595 | 78.67518 | 0.8005 | 26.6905 |
| Jiangsu **Lianyungang** Port | 2020 | 927111.699 | 5.3 | 8,596.33 | 86 | 832.48 | 30.26892 | 166.9568 | 49.31025 | 13.85042 | 184.5656 | 2.0642 | 24.0858 |
| Jiangsu **Lianyungang** Port | 2021 | 924760.294 | 5.9 | 11,994.04 | 87 | 880.44 | 33.73333 | 118.0631 | -29.2853 | 25.33612 | 95.38076 | 3.7293 | 22.5965 |
| **Solareast** Holdings | 2017 | 632124.166 | 2.283399 | 6356.786977 | 15.21197 | 366 | 72.07909 | 51122.84 | 142.1609 | 18.37577 | -76.156 | 1.5371 | 36.7722 |
| **Solareast** Holdings | 2018 | 580791.477 | 2.455099 | 7799.080023 | 15.39775 | 451 | 67.44186 | 49573.11 | -3.03137 | 14.10861 | -967.495 | -14.5727 | 32.6855 |
| **Solareast** Holdings | 2019 | 628325.227 | 2.426606 | 8168.196579 | 17.33032 | 383 | 56.13411 | 50683.8 | 2.240506 | 5.962589 | -117.61 | 2.7861 | 33.1994 |
| **Solareast** Holdings | 2020 | 620780.459 | 3.281722 | 11626.29025 | 13.92658 | 349 | 62.61869 | 49382.55 | -2.56739 | 5.247683 | 94.79112 | 5.2112 | 31.3813 |
| **Solareast** Holdings | 2021 | 627587.474 | 3.681052 | 15480.87774 | 15.74586 | 456 | 61.4862 | 48223.27 | -2.34755 | 18.70917 | 23.93004 | 6.0557 | 29.5078 |
| **Cisen** Pharmaceutical | 2017 | 474188.82 | 4.79321 | 14199.96262 | 40.52774 | 599 | 54.74074 | 18913.66 | -60.779 | -29.5571 | 70.40583 | 12.1431 | 51.0811 |
| **Cisen** Pharmaceutical | 2018 | 524839.047 | 8.560257 | 32598.13169 | 39.68983 | 691 | 60.45139 | 18898.08 | -0.08235 | 28.54201 | 37.56768 | 12.769 | 57.4131 |
| **Cisen** Pharmaceutical | 2019 | 553375.31 | 7.551089 | 31056.00777 | 42.421 | 792 | 62.588 | 20801.64 | 10.07275 | 8.001597 | 2.333576 | 11.7953 | 57.4332 |
| **Cisen** Pharmaceutical | 2020 | 616571.047 | 8.425999 | 30958.45445 | 37.01117 | 795 | 69.87638 | 20791.87 | -0.04699 | -10.665 | -15.0416 | 9.3296 | 57.2446 |
| **Cisen** Pharmaceutical | 2021 | 636371.506 | 8.839066 | 33434.585 | 18.2426 | 382 | 65.31503 | 21082.03 | 1.395561 | 2.951265 | -23.6552 | 6.7746 | 55.4632 |
| Anhui **Kouzi** Distillery | 2017 | 767635.712 | 0.387802 | 1397.113821 | 9.659715 | 88 | 23.83569 | 42417.39 | 21.2096 | 27.29398 | 42.146 | 23.8835 | 72.8951 |
| Anhui **Kouzi** Distillery | 2018 | 884225.521 | 0.353905 | 1510.80907 | 9.161148 | 83 | 23.75459 | 46396.46 | 9.380752 | 18.49522 | 37.62364 | 27.2422 | 74.3678 |
| Anhui **Kouzi** Distillery | 2019 | 950132.632 | 0.868658 | 4058.444088 | 11.89759 | 79 | 17.2378 | 44458.56 | -4.17682 | 9.443071 | 12.23634 | 26.105 | 74.9735 |
| Anhui **Kouzi** Distillery | 2020 | 980637.731 | 1.460464 | 5858.131504 | 7.038391 | 77 | 27.83007 | 43501.85 | -2.15192 | -14.1466 | -25.8378 | 17.9047 | 75.172 |
| Anhui **Kouzi** Distillery | 2021 | 1103089.35 | 1.461824 | 7350.925008 | 7.458803 | 86 | 28.8034 | 52895.31 | 21.59325 | 25.36568 | 35.37915 | 22.3023 | 73.9012 |
| Jiangsu Pacific **Quartz** | 2017 | 137965.332 | 2.989941 | 1683.698549 | 31.70103 | 123 | 40.58577 | 2826.046 | 21.20712 | 26.09482 | 33.19024 | 8.6454 | 36.9179 |
| Jiangsu Pacific **Quartz** | 2018 | 151521.893 | 3.478068 | 2202.65172 | 32.13429 | 134 | 36.74009 | 4983.974 | 76.35856 | 12.46203 | 32.05024 | 10.5321 | 43.7359 |
| Jiangsu Pacific **Quartz** | 2019 | 193808.714 | 4.185613 | 2604.865913 | 39.28571 | 176 | 38.03056 | 4829.307 | -3.10329 | -1.73053 | 14.60037 | 11.0712 | 43.58 |
| Jiangsu Pacific **Quartz** | 2020 | 217352.29 | 4.885763 | 3154.085419 | 37.25055 | 168 | 40.44843 | 7624.755 | 57.88507 | 3.732467 | 15.30921 | 10.7944 | 40.7365 |
| Jiangsu Pacific **Quartz** | 2021 | 244684.429 | 4.104407 | 3943.02577 | 34.46215 | 173 | 40.06385 | 7808.969 | 2.415995 | 48.81205 | 49.36775 | 13.6167 | 45.6265 |
| Shandong **Donghong** Pipe Industry | 2017 | 172602.01 | 2.81612 | 4242.428045 | 41.04478 | 110 | 24.38581 | 8190.615 | 4.887282 | 56.81377 | -56.7418 | 10.6526 | 22.1439 |
| Shandong **Donghong** Pipe Industry | 2018 | 188652.794 | 2.633732 | 4312.678036 | 40.60403 | 121 | 26.32509 | 7992.634 | -2.41717 | 8.695639 | 27.1028 | 10.3549 | 22.5818 |
| Shandong **Donghong** Pipe Industry | 2019 | 208806.854 | 2.904241 | 5339.655164 | 36.38614 | 147 | 30.03717 | 9753.768 | 22.03446 | 12.28071 | 30.3609 | 12.3739 | 25.9902 |
| Shandong **Donghong** Pipe Industry | 2020 | 286843.375 | 2.585658 | 6186.291454 | 41.48936 | 156 | 26.46024 | 9597.723 | -1.59984 | 30.13039 | 58.15256 | 17.3845 | 26.2271 |
| Shandong **Donghong** Pipe Industry | 2021 | 346825.747 | 2.424475 | 5355.487818 | 46.2908 | 156 | 26.32813 | 12964.29 | 35.07673 | -7.67445 | -58.2309 | 6.7121 | 19.4715 |
| **Novoray** Corporation | 2019 | 102372.652 | 4.07007 | 1283.297531 | 29.03226 | 36 | 36.47059 | 1021.39 | -92.1215 | -85.726 | -43.8538 | 12.2885 | 46.309 |
| **Novoray** Corporation | 2020 | 109256.873 | 4.894598 | 1978.41327 | 21.31148 | 39 | 50.13699 | 1003.701 | -1.73181 | 28.19601 | 48.49222 | 11.9259 | 42.8397 |
| **Novoray** Corporation | 2021 | 130490.215 | 5.611607 | 3505.624775 | 27.97927 | 54 | 40.88983 | 5301.89 | 428.2339 | 54.55327 | 55.85432 | 16.8024 | 42.4619 |
