# Supplementary material for: Analyzing the innovation ability of listed companies in the core area of the Huaihai economic zone
Source: PLoS One. 2023 May 2;18(5):e0285181. doi: 10.1371/journal.pone.0285181 (PMC10153750; doi:10.1371/journal.pone.0285181)
Supplement: Attachment — Submitted filename: Response to Reviewers.docx [file pone.0285181.s002.docx]

Reviewer #

Title

1. Instead of using word “Research” use some other appropriate word describing the main objective of the study such as “Analyzing the innovative ...” Or
2. “Identifying determinants of innovative……..” or “Factors affecting innovative …….” as at end of the introduction while describing objectives author(s)

The title has been revised in accordance with these recommendations as follows: “Analyzing the innovation ability of listed companies in the core area of the Huaihai economic zone.”

Abstract
1. Add the importance/justification of the study i.e. why this research has been undertaken in the beginning and then describe the objectives of the study.

2. Add importance/justification the choice of the “Entropy Weight Analysis” method and description of source of the data collection.

3. In the last 2-3 sentences of the abstract, actual results, concluding statement and main recommendation(s) should be provided.

According to these recommendations, the abstract has been modified in three ways. First, the significance and the objective of the research have been highlighted. Second, the selection of methods were included, and the sources of data collection have been added. Third, conclusions and main recommendations have been provided at the end of the abstract.

Introduction
1. Justification of the study is lacking. Why the objectives of the study are important for conducting this research. Problem statement should clearly be defined and objectives should be described more clearly.
2. Avoid long sentences and use proper punctuation, commas and semicolons, to separate the parts of a sentence so that reader can easily understand what the author intends to say.
 In accordance with these recommendations, the introduction has been revised. A statement of the significance and objectives of the study has been added, and the language and content have been polished.

Review of Literature
1. All/most of the review should be comprehended. Review of about nine studies is added while Author(s) stated in the beginning of the last paragraph of the review of literature that comprehensive research is available and also mentioned the various methods e.g. factor analysis. So, all such literature should be comprehended.
2. If possible, provide the conceptual framework of the study
 The literature review has been polished and modified to make the content easier to understand; it also provides a conceptual framework for the research.

Methodology (Study Design)
1. Instead of study design as a main heading, heading such as methodology or data and methods etc can be given and study design could be the sub-headings
 The main heading has been revised as suggested.

2. Write some intro of study design before the heading “Construction of the innovation capability evaluation index system” to depict what is going to be written in upcoming sub-headings
 According to the recommendations, the content about the research design has been revised, and the sample sources and research methods have been introduced.

3. A heading, “Theoretical Background”, may be added for presenting theories used / theoretical literature.
 The Theoretical Background heading has been added as suggested.

4. Data collection and analytical methods (Entropy Method) should be backed by the literature and justification should be provided that why this is preferred over other methods such as factor

Because the research object of this paper is panel data, the entropy method is more suitable than the factor analysis method. In addition, to conduct more comprehensive and scientific research, this paper also adds the TOPSIS-Entropy combined method to compare and verify the previous analysis results.

Results and Discussion (Evaluation of the innovation ability of listed companies in the core area of the Huaihai economic zone)
1. Instead of “Evaluation of the innovation ability of listed companies in the core area of the Huaihai economic zone”, Results and Discussion” heading may be used and sub-headings under this main headings could be used
 The heading has been revised as suggested.

2. Table 6 onward and figure 2 onward, only the findings re described and expected reasons behind these findings are lacking. It is suggested that some logics behind these findings should be added
 The survey results and the expected reasons for these findings have been added in accordance with these recommendations.

3. Top companies with high input and high output should be explained well so that they can serve as role model.
 According to these recommendations, the analysis of typical model enterprises with high input and high output has been further explained (e.g., Hengrui).

4. Comparison of this study with the earlier studies is not added. Such discussion must be added so that reader can have a clear idea that how the findings of this study support or contradict the findings of earlier studies.
 Due to the lack of research on the innovation ability of the listed enterprises in the Huaihai Economic Zone, there is no way to compare our research with earlier studies. Therefore, this paper adds the TOPSIS-Entropy combined method as a comparative supplementary analysis method to evaluate the Huaihai Economic Zone more scientifically and comprehensively.

Suggestions
Suggestions must come from the findings of the data analysis of the study. In this regard, improve suggestion 2 and 3.
This part has been modified and improved according to these suggestions.

Reviewer #2: This paper is a good effort to analyze innovation ability of companies in the Huaihai region. Methodolgoy of the entropy method is appropriate to address the objective of this study. In upcoming research on this aspect relying on just one method (entropy method) would not suffice. A mix method approach would provide better insight.

The paper has been modified and improved according to these suggestions, and the TOPSIS-Entropy combined method has been added.
